# Spatiotemporal dynamics of urban green spaces and human–wildlife conflicts in Tokyo

**DOI:** 10.1038/srep30911

**Published:** 2016-08-02

**Authors:** Tetsuro Hosaka, Shinya Numata

**Affiliations:** 1 Department of Tourism Science, Graduate School of Urban Environmental Sciences, Tokyo Metropolitan University, 1-1 Minami-Osawa, Hachioji, Tokyo 192-0397, Japan

## Abstract

Although urban green spaces are increasingly important both for humans and wildlife, an increase in urban green spaces may also increase human–wildlife conflicts in urban areas. However, few studies have examined the relationship between the size of green spaces and the level of conflicts with wildlife in multiple taxa, including invertebrates and vertebrates. To better understand current pest statistics and predict changes that will occur as the area of green spaces increases, we analysed a dataset compiling the number of pest consultations in 53 metropolitan districts in Tokyo over a 20-year period and its relationships with the area of green space. Stinging insects (e.g., wasps) made up over 50% of pest consultations, followed (in order) by rats and other nuisance animals (e.g., snakes). The number of consultations per unit population did not correlate, or was even negatively correlated, with the proportions of green spaces (mainly forest) for many indoor pests, but did positively correlate for some outdoor pests, such as wasps and snakes. Therefore, wasps and snakes can increase when urban green spaces increase. Because even minor nuisances are relevant for urban lifestyles, considerations of ways to minimise conflicts with wildlife are critical for urban green space management.

As the urban population exceeds 50% of the world population and is increasing^1^, the quantity and quality of green spaces in urban areas have become key elements for sustaining the quality of human life and biological diversity in urban areas^2^. The ecosystem services provided by urban green spaces include air filtering, micro-climate regulation, noise reduction, rainwater drainage, sewage treatment, and recreational and cultural values^3^. The creation of larger or more natural green spaces in the city provides valuable habitat for many plants and animals in urban areas^4^.

However, some products of natural ecosystems may be negative or harmful, and are often termed “ecosystem disservices”^5^,^6^. Ecosystem disservices in urban areas include allergen emissions, damage caused to human infrastructures and people by fallen branches, fear of and stress related to shaded green areas during darkness, and larger populations of pests and nuisance animals^7^. Negative aspects of urban wildlife have been well documented, including nuisance effects and property damage, involvement in road vehicle collisions, and dispersal of zoonotic and vector-borne diseases^8^. Human–wildlife interactions occur most frequently in the intermediate levels of development, often in the vicinity of natural patches of habitat or green spaces^8^,^9^. Although most studies of human–wildlife (i.e., wild animals including insects) conflicts have focused on conflicts with large mammals^10^, urban residents are usually more annoyed by insect pests and smaller nuisance animals (hereafter, these two categories of animals are referred to as pests). However, few urban wildlife studies have covered a wide range of animals, including arthropods, reptiles, birds, and mammals^11^,^12^, and the full picture of human–wildlife interactions and their spatiotemporal dynamics have rarely been documented. In this study, we used the number of pest consultations in Tokyo as a measure of urban human–wildlife conflicts and addressed the following questions:

(1) What are the major current pests in Tokyo?

(2) Is the number of pest consultations increasing or decreasing?

(3) Is the number of consultations per population related to green space size?

## Methods

### Study site

Tokyo, the capital city of Japan, is one of the largest cities in the world. The human population of >13 million people lives in its metropolitan area of 2,200 km^2^. Mainland Tokyo comprises 53 districts (i.e. wards, cities, towns and villages) managed by local governments. The level of urbanisation differs considerably between the eastern and western sectors. The eastern sector, the city centre, is highly urbanised and contains little forested (0.1% of total area) or agricultural (1.1%) land^13^. The western sector, however, is an urban fringe with considerable areas of forest (48.7%) and agricultural (5.5%) land.

Although the total area of green space in Tokyo has decreased continuously since the 1960s through attritions in forested and agricultural land, the rate decline has been slowing through time (Fig. S1). Conversely, the area of urban park land has increased continuously; it slightly exceeded the area of agricultural lands in 2012. After the Convention on Biological Diversity at Nagoya in 2010, the Tokyo Metropolitan Government and individual local governments established strategies for urban biodiversity conservation. They planned to increase the size and value of green spaces for local biodiversity through improvements in their quality and connectivity^14^. For example, the Tokyo Metropolitan Government planned to create 1000 ha of new green spaces within 10 years^14^.

### Data

We used data on pest consultations provided by local residents to local governments and health centres in 53 Tokyo districts between 1995 and 2014. These data have been compiled by the Tokyo Metropolitan Government in every fiscal year (April to March). Experienced consultants for local governments and health centres categorised animals under consultation into 12 groups (Table 1) and 82 sub-groups (Table S1) during hearings and investigations of samples. We included the numbers of consultations for unidentified pests in the “others” subgroup of each group category. When even the group categories of the unidentified animals were uncertain, we included them in the “others” group. We collected data on the population and land use in each district in 2011 from the website of the Statistics Division, Bureau of General Affairs^15^.

### Data analysis

To identify the major current pests in Tokyo, we pooled available data on the numbers of pest consultations compiled over the previous 5 years (2010–2014) and calculated the proportions of consultations relating to each of the pest group and sub-group categories.

To identify increases and decreases in the numbers of consultations over a 20-year period for each pest, we calculated Spearman’s rank correlation coefficients (rho) for the relationships between the numbers of consultations per pest group/subgroup and the calendar year (n = 20). We combined the numbers of consultations across all districts.

We performed spatial simultaneous auto-regressive lag model estimations using the *spdep* package^16^ and R ver. 3.1.2 software^17^ to test whether the number of consultations significantly correlated with the proportions of green space in each district. The lag model considers the spatial auto-correlation of the response variable using the maximum likelihood estimation of spatial simultaneous autoregressive lag and the spatial Durbin model^16^. We measured the spatial adjacency of the districts using Delaunay triangulation. Since the number of consultations in each district was strongly correlated with district population size (total number of consultations *vs.* population size: rho = 0.85, P < 0.001), we used the number of consultations per 10,000 population members in each district (n = 53) over the previous five years as a response variable in the model, rather than the number of consultations. The response variable was log-transformed prior to the analysis to improve normality. Normality was checked using normal Q-Q plots. We selected the three major types of green space in the study region: forest, agricultural land and urban park. We used the proportions of each green space category in the total area of each district as explanatory variables in the model. We standardised the explanatory variables to calculate standardised regression coefficients. We calculated 95% confidence intervals (CI) for each regression coefficient based on the mean and standard error of each. We evaluated the statistical significances of the coefficients by determining whether the 95% CIs overlapped with zero.

## Results

### Major pests in Tokyo

For a total of 162,053 consultations conducted between 2010 and 2014, stinging insects (e.g., wasps and bees), rats, nuisance animals (e.g., snakes, crows and civets) and blood-sucking insects (e.g., mosquitoes and lice) were the four most important groups among the 12 pest group categories (Table 1). Stinging insects alone accounted for 50% of all consultations. Among the 82 sub-groups, consultations on stinging paper wasps (Polistinae) and hornet wasps (Vespinae) were the most frequent (40% of the total) (Table S1).

### Temporal patterns of pest consultations

**Figure 1:**
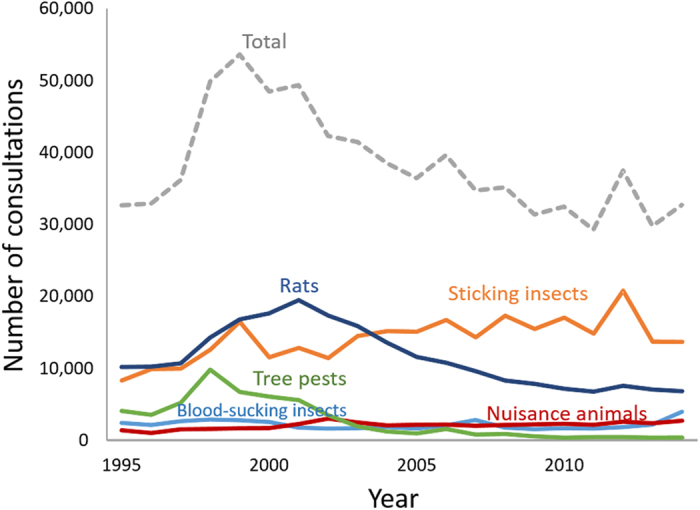
Number of consultations on the five major pest groups (stinging insects [orange], rats [blue], nuisance animals [red], blood-sucking insects [light blue] and tree pests [green]) in Tokyo during the period 1995–2014. The grey broken line shows the total number of pest consultations.

Consultation numbers peaked in 1999 and decreased gradually from 2000 until 2014 (Fig. 1). Among the twelve groups of pests, consultations on two (stinging insects and nuisance animals) increased significantly, consultations on six (rats, nuisance insects, sanitary pests, mites, tree pests and food pests) decreased significantly and consultations on four (blood-sucking insects, poisonous caterpillars, wood pests and others) did not change significantly over 20 years (Table 1). Rats were the most important pests measured by the numbers of consultations until 2003, but ranked second to stinging insects in the last half of the 20-year study period (Fig. 1).

Among the 82 subgroups, consultations on 17 increased significantly, 34 decreased significantly and 31 did not change significantly over the 20-year period (Table S1). Among stinging insects, the numbers of consultations on hornet wasps and carpenter bees increased significantly over time, but the number of consultations for paper wasps and honey bees did not (Table S1). The number of consultations for the rat subgroups did not change significantly over time. Among nuisance animals, the numbers of consultations on doves, snakes and others increased significantly over time, but those on millipedes and centipedes decreased significantly.

### Spatial patterns of pest consultation

**Figure 2:**
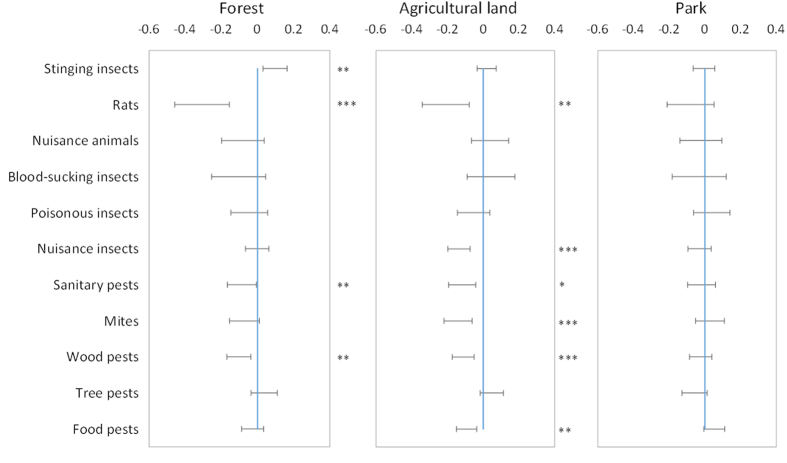
Confidence intervals (95%) of regression coefficients (estimated by spatial simultaneous auto-regressive lag models) for the three green spaces (forest, agricultural and park land) used to explain the numbers of consultations per population for each pest group. Blue lines indicate zero. The levels of significance were assessed with z-tests: *P < 0.05; **P < 0.01; ***P < 0.001.

Among 11 pest groups, the regression coefficients for the relationships with green space (forest, agricultural land and park) were significantly positive only for stinging insects, significantly negative for six groups (mites, sanitary pests, nuisance insects, food pests, tree pests and rats) and not significant for four groups (nuisance animals, blood-sucking insects, poisonous insects and tree pests) (Fig. 2). The number of consultations for stinging insects per unit population consultations was greater in districts with high proportions of forested area. The significantly negative coefficients for some pest groups indicate that such consultations were more frequent in highly urbanised districts, since the proportions of forested and agricultural land were negatively correlated with the proportions of residential area (forest: r = −0.69, P < 0.001, agricultural land: rho = −0.31, P < 0.05).

Among the 82 subgroups, the regression coefficients for the relationships with forest and agricultural land areas were significantly positive for only eight subgroups (hornet wasp, carpenter bee, wingless wasp, millipede, snake and Japanese copper head, hard tick and caterpillar), significantly negative for 29 subgroups, and not significantly positive nor negative for the remaining 45 subgroups (Fig. S2).

## Discussion

Our 20-year dataset indicates that the numbers of consultations decreased for many indoor pests such as rats and mites, a trend that might be attributable to improvement in sanitary conditions, changes in housing structures and widespread use of pesticides in modern homes. Conversely, the number of consultations on outdoor pests such as stinging insects and nuisance animals increased over time; at the end of the study period, they ranked first and third in the number of consultations. Hornet wasps are particularly important, being the most harmful animals in Japan; 13–45 people were killed annually by hornets in 1989–2013, a number that exceeds deaths attributed to other animals^18^. Of the 53 local governments, 24 partially or totally compensated local residents for expenses incurred in the removal of hornet nests from their houses. Note, however, that the increased number of these consultations would not be due to the changes in green space area, because the area in Tokyo did not increase in the 20 years (Fig. S1), but would be due to other factors such as a decrease in public tolerance, as discussed below.

In contrast to our expectations, the size of green spaces was not significantly correlated, or was even negatively correlated, with the number of consultations for many of the pest groups and subgroups, particularly for indoor pests. The negative correlations likely reflect the positive correlations between the number of consultations and housing areas, rather than the effects of green spaces. It is natural to consider that these pests were more abundant where human density is higher, irrespective of the amount of outdoor green space. Even a small green space could be sufficient to sustain a population of some small pests, such as mosquitoes.

On the other hand, the positive association between the size of green space and the number of consultations for some outdoor pests (e.g., hornets and snakes) suggests that conflicts with these pests may increase if more green spaces are created in urban areas in the future. Previous studies also provide evidence that an increase in green spaces can increase human–wildlife conflicts in urban areas. For example, the removal of wasp nests by the Korean 119 Rescue Service was concentrated around green spaces in the city of Seoul^19^. Kretser *et al*.^9^ reported that in New York human–wildlife conflicts were most frequent in exurban landscapes with intermediate housing densities, where human–wildlife interactions occurred more often than in urban and rural landscapes. Our recent study also demonstrated that hornets were more abundant in greener urban areas^20^.

The frequency of pest consultations may also increase with declining public tolerance of wildlife. Urban residents were more sensitive to social wasps and more frequently called the Korean 119 Rescue Services to remove them than were rural residents in Seoul^19^. Moreover, the tolerance of problems caused by wildlife in New York declined in the period 1984–1996, particularly among young urban residents^21^. The increasing number of consultations for some pests in Tokyo might therefore be related to declining public tolerance. Media representations have a particularly strong influence on the attitudes of urban residents toward nature^22^. In Japan, the TV and radio services broadcast more than 30 shows on hornet wasps in 6 months, often illustrating the extermination of hornet nests, with emphasis on the dangerous nature of these insects^23^. These shows are likely to make urban residents more sensitive to hornets and increase the number of consultations. The negative correlations between many pests and green spaces may be partly due to greater sensitivity toward animals among urban residents, who often lack experiences with wildlife^24^.

A few caveats are to be mentioned. Our data based on official consultations is biased towards serious cases, and thus there are many more cases that were not reported. Therefore, our data might actually underestimate the level of nuisance caused by urban wildlife. Our study also did not include data allowing for more detailed analyses, such as on the potential effects of different maintenance strategies for each urban green spaces. Such analyses will provide more detailed suggestions regarding green space management. The effects of green walls and rooftops on human–wildlife interaction should also be assessed because these types of green space are increasingly common in urban areas^4^,^25^.

In conclusion, our study suggests that increasing green spaces in urban areas will not increase most pest animals, but may increase some outdoor pests (e.g., hornets and snakes), which may decrease public support for biodiversity-friendly green spaces in urban areas and increase government costs for management; the extermination of hornet nests normally costs US$100–500 per nest in Japan. For sustainable green space management, urban planners need to consider the responses of favourable species to be conserved and also those of pests and unfavourable species in urban areas. Furthermore, the increase in consultations on wasps and nuisance animals suggests that public tolerance of these animals might have decreased over the 20 years. The tolerance and attitudes toward wildlife vary among residents depending on the values^26^, socio-demographic characteristics, and experiences of the population^10^. A lack of experience with wildlife might be an essentially contributor to declines in tolerance and increases in fears toward wildlife among urban residents^27^. Hence, urban green planning should also include communication on the relevant manners about how to interact with wildlife to avoid problems caused by them.

## Additional Information

**How to cite this article**: Hosaka, T. and Numata, S. Spatiotemporal dynamics of urban green spaces and human-wildlife conflicts in Tokyo. *Sci. Rep.* **6**, 30911; doi: 10.1038/srep30911 (2016).

## Supplementary Material

Supplementary Information

## Figures and Tables

**Table 1:** Number of consultations for each pest group in Tokyo in the period 2010‒2014.

Pest group	N consultation (%)		Rho	
Stinging insects	80312	(49.6)	0.623	^**^
Rats	35670	(22.0)	−0.705	^***^
Nuisance animals	12141	(7.5)	0.717	^***^
Blood-sucking insects	11318	(7.0)	−0.257	
Poisonous insects	3363	(2.1)	−0.411	
Nuisance insects	3327	(2.1)	−0.908	^***^
Sanitary pests	2911	(1.8)	−0.961	^***^
Mites	2864	(1.8)	−0.884	^***^
Wood pests	2801	(1.7)	−0.168	
Tree pests	1900	(1.2)	−0.914	^***^
Food pests	1368	(0.8)	−0.846	^***^
Others	4078	(2.5)	0.177	
Total	162053	(100.0)	−0.511	^*^

Spearman’s rank correlation coefficients (rho) for the relationships between the number of consultations and calendar years in the period 1995‒2014 are provided: ^*^P < 0.05; ^**^P < 0.01; ^***^P < 0.001.
