# Supplementary material for: Spatiotemporal dynamics of urban green spaces and human–wildlife conflicts in Tokyo
Source: Sci Rep. 2016 Aug 2;6:30911. doi: 10.1038/srep30911 (PMC4969584; doi:10.1038/srep30911)
Supplement: Supplementary Information [file srep30911-s1.pdf]

Supplementary Information

Title: Spatiotemporal dynamics of urban green spaces and human-wildlife conflicts in Tokyo

Authors: Tetsuro Hosaka, Shinya Numata

Table S1. Number of consultations on each pest subgroup in Tokyo in the period 2010–2014; Spearman's rank correlation coefficients (rho) for the relationships between the number of consultations and calendar years during the period 1995–2014 are provided: *, $P < 0.05$; **, $P < 0.01$; ***, $P < 0.001$.

Group	Subgroup	Nomenclature	Family	N consultation	(%)	rho	
Stinging insects	Paper wasp	Polistinae	Vespidae	37023	(22.8)	0.319	
	Hornet wasp	Vespinae	Vespidae	26937	(16.6)	0.841	***
	Honey bee	*Apis* spp.	Apidae	1022	(0.6)	-0.337	
	Bumble bee	*Bombus* spp.	Apidae	343	(0.2)	-0.116	
	Carpenter bee	*Xylocopa appendiculata circumvolans*	Apidae	417	(0.3)	0.918	***
	Wingless wasp	*Cephalonomia gallicola*	Bethylidae	149	(0.1)	-0.752	***
	Other Hymenoptera			14138	(8.7)	0.415	
	Horsefly	Tabanidae	Tabanidae	48	(0.0)	0.597	**
	Others			235	(0.1)	0.214	
Rats	Brown rat	*Rattus norvegicus*	Muridae	1090	(0.7)	-0.828	***
	Black rat	*Rattus rattus*	Muridae	10659	(6.6)	0.071	
	House mouse	*Mus musculus*	Muridae	72	(0.0)	-0.605	**
	Others			23849	(14.7)	-0.800	***
Nuisance animals	Millipede	Diplopoda		276	(0.2)	-0.475	*
	Centipede	Chilopoda		198	(0.1)	-0.644	***
	Spider	Araneae		713	(0.4)	0.382	
	House centipede	Scutigeromorpha		36	(0.0)	-0.633	***
	Crow	*Corvus* spp.	Corvidae	3548	(2.2)	-0.212	
	Dove	*Columba livia*	Columbidae	1753	(1.1)	0.991	***
	Snake	Serpentes		1215	(0.7)	0.810	***

	Japanese copper head	*Gloydius blomhoffii*	Viperidae	49	(0.0)	0.335	
	Others			4353	(2.7)	0.974	***
Blood-sucking insects	Head louse	*Pediculus capitis*	Pediculidae	5222	(3.2)	0.680	***
	Crab louse	*Pthirus pubis*	Pthiridae	23	(0.0)	-0.858	***
	Human louse	*Pediculus humanus*	Pediculidae	69	(0.0)	0.100	
	Other louse			265	(0.2)	0.804	***
	Cat flea	*Ctenocephalides felis*	Pulicidae	320	(0.2)	0.974	***
	Other flea			205	(0.1)	-0.447	*
	Tiger mosquito	*Aedes* spp.	Culicidae	1169	(0.7)	-0.704	***
	House mosquito	*Culex* spp.	Culicidae	147	(0.1)	0.498	*
	Other mosquito			2394	(1.5)	0.597	**
	Bed bug	*Cimex lectularius*	Cimicidae	1446	(0.9)	0.903	***
	Others			58	(0.0)	0.192	
Poisonous insects	Tea tussock moth	*Euproctis pseudoconspersa*	Lymantriidae	1166	(0.7)	-0.594	**
	Tussock moth	*Artaxa subflava*	Lymantriidae	17	(0.0)	-0.933	***
	Slug moth	Limacodinae	Limacodinae	224	(0.1)	0.204	
	Eilema fuscodorsalis	*Eilema fuscodorsalis*	Arctiidae	17	(0.0)	-0.126	
	Other moth			1881	(1.2)	0.009	
	Others			58	(0.0)	0.285	
Nuisance insects	Nonbiting midges	Chironomidae	Chironomidae	401	(0.2)	-0.901	***
	Psocids	Psocoptera		257	(0.2)	-0.899	***
	Ants	Formicidae	Formicidae	1110	(0.7)	0.229	
	Winged ants	Formicidae	Formicidae	747	(0.5)	0.770	***
	Stink bug	Hemiptera	Hemiptera	127	(0.1)	-0.239	
	Others			685	(0.4)	-0.113	
Sanitary pests	German cockroach	*Blatella germanica*	Blattellidae	372	(0.2)	0.601	**
	Smoky-brown cockroach	*Periplaneta fuliginosa*	Blattidae	146	(0.1)	0.689	***
	Other cockroach			678	(0.4)	-0.910	***

	Maggot			169	(0.1)	-0.387	
	Other Diptera			1107	(0.7)	-0.950	***
	Psychodid	Psychodidae	Psychodidae	406	(0.3)	-0.318	
	Others			33	(0.0)	0.090	
Mites	Cheyletid mites	Cheyletidae	Cheyletidae	175	(0.1)	-0.869	***
	House dust mite	*Ornithonyssus bacoti*	Macronyssidae	457	(0.3)	0.395	
	Northern fowl mite	*Ornithonyssus sylviarum*	Macronyssidae	48	(0.0)	0.129	
	Dermanyssus hirundinis	*Dermanyssus hirundinis*	Dermanyssidae	1	(0.0)	-0.016	
	Itch mite	*Sarcoptes scabiei var. hominis*	Sarcoptidae	119	(0.1)	-0.370	
	Hard tick	Ixodidae	Ixodidae	234	(0.1)	-0.058	
	Dust mites	Mealia	Pyroglyphidae	180	(0.1)	0.263	
	Acarid mites	Acaridae	Acaridae	78	(0.0)	-0.851	***
	Erythraeid mites	Erythraeidae	Erythraeidae	282	(0.2)	-0.576	**
	Other mites			1213	(0.7)	-0.930	***
	Chiggers	Trombiculidae	Trombiculidae	15	(0.0)	-0.381	
	Others			62	(0.0)	0.197	
Wood pests	Japanese termite	*Reticulitermes speratus*	Rhinotermitidae	884	(0.5)	0.689	**
	Other termite			1732	(1.1)	-0.489	*
	Powder-post beetle	Lyctidae	Lyctidae	83	(0.1)	-0.490	*
	Others			102	(0.1)	0.396	
Tree pests	Caterpillar	Lepidoptera		1486	(0.9)	-0.871	***
	Leaf beetle	Chrysomelidae	Chrysomelidae	23	(0.0)	-0.555	*
	American fall webworm	*Hyphantria cunea*	Arctiidae	85	(0.1)	-0.932	***
	Scale insects	Coccoidea		22	(0.0)	-0.798	***
	Others			284	(0.2)	-0.69	**
Food pests	Drugstore beetle	*Stegobium paniceum*	Anobiidae	195	(0.1)	-0.936	***
	Cigarette beetle	*Lasioderma serricorne*	Anobiidae	323	(0.2)	-0.777	***
	Black carpet beetle	*Attagenus japonicus*	Dermestidae	141	(0.1)	-0.819	***
	Varied carpet	*Anthrenus verbasci*	Dermestidae	134	(0.1)	0.491	*

beetle						
Maize weevil	*Sitophilus zeamais*	Dryophthoridae	43	(0.0)	-0.922	***
Spider beetle	Ptinidae	Ptinidae	29	(0.0)	-0.550	*
Silverfish	Lepismatidae	Lepismatidae	97	(0.1)	-0.492	*
Pyralid moths	Pyralidae	Pyralidae	138	(0.1)	-0.011	
Others			268	(0.2)	-0.788	***

Figure S1. Changes in area for forests (△), agricultural lands (+), park (×) and total of these (○) in Tokyo in the period 1965–2013. Data source: Kyutokenshi shunokaigi (http://www.tokenshi-kankyo.jp/green/shiryou.html).

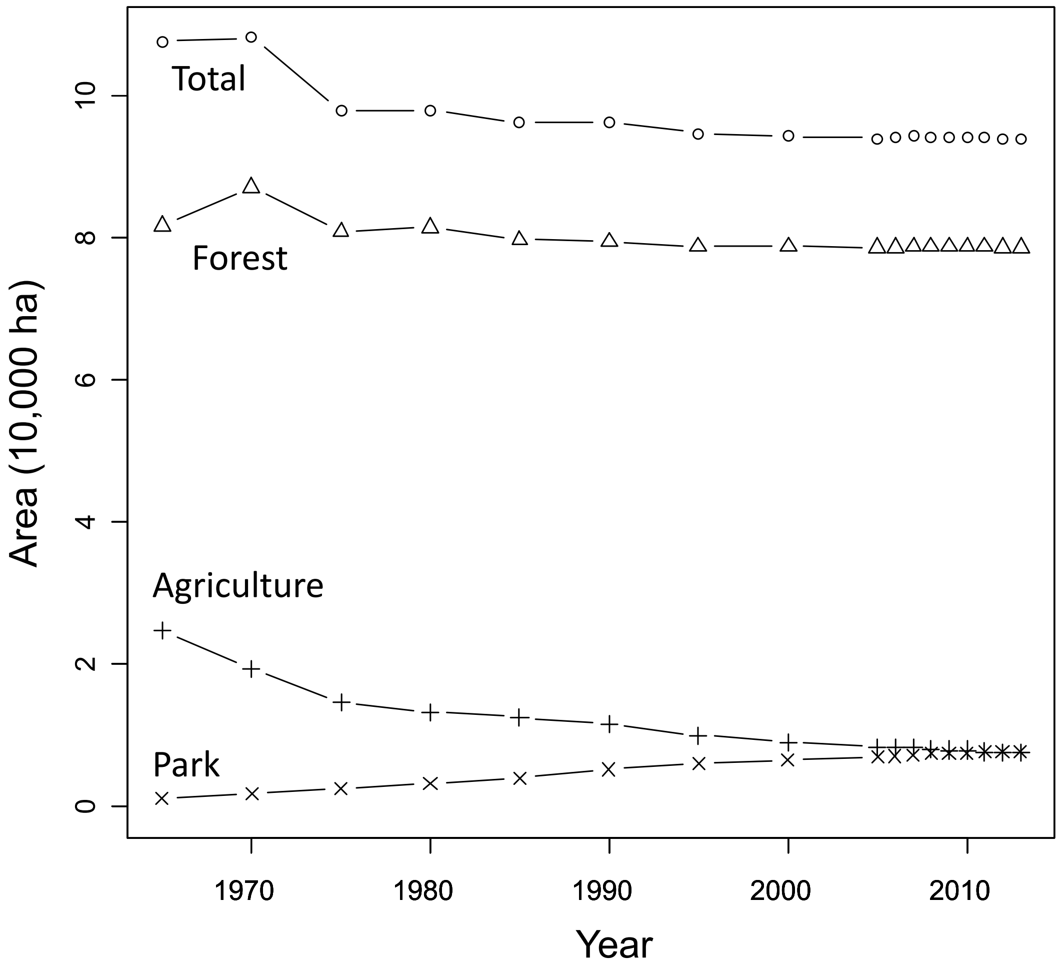

Figure S2. Confidence intervals (95%) of regression coefficients (estimated by spatial simultaneous auto-regressive lag models) for the three green spaces (forest, agricultural and park land) to explain the numbers of consultations per population for each pest subgroup. Blue lines indicate zero. The levels of significance were assessed with z-tests: *, $P < 0.05$; **, $P < 0.01$; ***, $P < 0.001$.

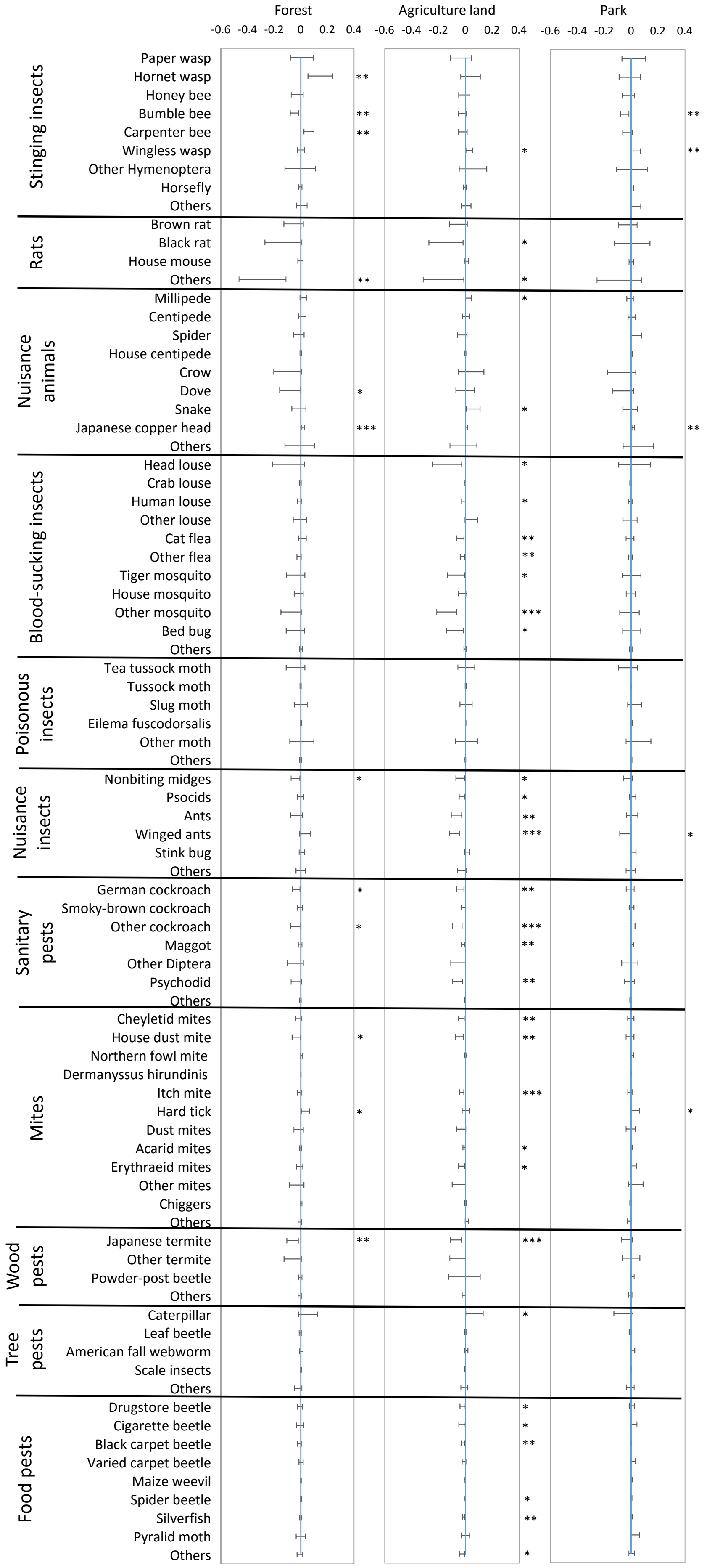
Forest
Agriculture land
Park
-0.6 -0.4 -0.2 0 0.2 0.4
Stinging insects
Paper wasp
Hornet wasp
Honey bee
Bumble bee
Carpenter bee
Wingless wasp
Other Hymenoptera
Horsefly
Others
Rats
Brown rat
Black rat
House mouse
Others
Nuisance animals
Millipede
Centipede
Spider
House centipede
Crow
Dove
Snake
Japanese copper head
Others
Blood-sucking insects
Head louse
Crab louse
Human louse
Other louse
Cat flea
Other flea
Tiger mosquito
House mosquito
Other mosquito
Bed bug
Others
Poisonous insects
Tea tussock moth
Tussock moth
Slug moth
Eilema fuscodorsalis
Other moth
Others
Nuisance insects
Nonbiting midges
Psocids
Ants
Winged ants
Stink bug
Others
Sanitary pests
German cockroach
Smoky-brown cockroach
Other cockroach
Maggot
Other Diptera
Psychodid
Others
Mites
Cheyletid mites
House dust mite
Northern fowl mite
Dermanyssus hirundinis
Itch mite
Hard tick
Dust mites
Acarid mites
Erythraeid mites
Other mites
Chiggers
Others
Wood pests
Japanese termite
Other termite
Powder-post beetle
Others
Tree pests
Caterpillar
Leaf beetle
American fall webworm
Scale insects
Others
Food pests
Drugstore beetle
Cigarette beetle
Black carpet beetle
Varied carpet beetle
Maize weevil
Spider beetle
Silverfish
Pyralid moth
Others
